# Serum Sclerostin, Body Composition, and Sarcopenia in Hemodialysis Patients with Diabetes

**DOI:** 10.1155/2020/4596920

**Published:** 2020-02-10

**Authors:** Maria Carolina Medeiros, Natalia Rocha, Elba Bandeira, Isabel Dantas, Conceição Chaves, Mario Oliveira, Francisco Bandeira

**Affiliations:** ^1^Division of Endocrinology and Diabetes, Agamenon Magalhaes Hospital, University of Pernambuco Medical School, Recife, Brazil; ^2^Uninefron-Unidade Nefrológica, Recife, Brazil; ^3^Department of Nutrition, Federal University of Pernambuco, Recife, Brazil

## Abstract

Sclerostin (Scl) is an osteoblast-inhibiting glycoprotein that is secreted mainly by osteocytes and is regulated by hormonal changes and skeletal loading. Decreased physical function and high serum Scl concentrations have been reported in chronic renal failure patients but little is known to date about the differences between diabetic and non-diabetic patients on hemodialysis who are susceptible to both sarcopenia and bone fragility. *Objective*.To determine the prevalence of sarcopenia and its association with serum Scl concentrations and metabolic parameters in 92 patients on hemodialysis. Anthropometric data and physical performance were evaluated in this study. Blood samples were collected for Scl, glucose, cholesterol, triglycerides, calcium, phosphate, PTH, and 25 OH-vitamin D measurements. Lean mass was evaluated using multifrequency electro-bioimpedance after dialysis session. *Results*. Mean age was 63.3 ± 13.6 years, 63% of patients were male, and 44.6% had diabetes. Mean body mass index (BMI) was higher in diabetics (26.6 ± 5.2 vs. 24.1 ± 3.7; *p*=0.01) and there were no differences in gait speed and handgrip strength between diabetic and non-diabetic subjects. A low skeletal muscle mass index (SMI) was identified in 65.2% of the participants, and among them 76.7% were men and 36.7% were diabetics. Mean serum Scl was 86.9 ± 39.0 pmol/L, which was higher in men (94.6 ± 41.7; *p*=0.017), in those individuals with low SMI (94.9 ± 40.7; *p* < 0.001), and in diabetics (97.2 ± 46.6; *p* < 0.003). After multivariate analysis and adjustments for potential confounders, high serum Scl was independently associated with low SMI and with the presence of diabetes. The following variables correlated positively with diabetes: blood pressure; BMI; waist circumference; waist/hip ratio; plasma glucose; serum Scl; and fat mass. *Conclusions*. We found higher serum Scl concentrations in hemodialysis patients with diabetes and these were inversely related to muscle mass.

## 1. Introduction

Sarcopenia can be defined as a gradual and generalized loss of muscular mass and muscular strength, which may be caused by advanced age, chronic diseases, physical inactivity, use of some medicines, and/or nutritional deficit. As a consequence of protein loss and metabolic damage caused by uremia, chronic kidney disease can result in reduced muscle synthesis and sarcopenia. Diabetes mellitus (DM) is one of the main causes of chronic kidney disease (CKD) and is also associated with increased risk of sarcopenia, which may be related to peripheral neuropathy due to reduced neuronal stimulation, insulin resistance, a pro-inflammatory condition, mitochondrial dysfunction, and oxidative injury [1].

Besides sarcopenia, individuals with CKD present disturbances in bone metabolism. Kidney osteodystrophy causes loss of bone quality and strength, leading to fractures as well as to increased rates of morbidity and mortality [2]. Higher risk of fractures has been reported in individuals with DM, and osteoporotic fractures are considered to be a complication of DM [3].

A study carried out on mice showed that type 2 DM has harmful effects on trabecular bone architecture and cortical bone geometry due to low bone formation and high bone reabsorption [4]. However, humans with type 2 diabetes have a high risk of fractures in spite of normal bone mineral density and bone turnover [5].

Studies have been performed to investigate the relationship between muscle and bone mass and the influence they have on each other. In this context, Jackowski et al. [6] in their study reported that the quantity of lean body mass accumulated in individuals during adolescence until they become young adults has a positive influence on the structural strength of the adult proximal femur bone.

Further evidence of the relationship between muscle and bone was identified by observing that sclerostin can partially account for bone loss and the deterioration of bone structure in older obese individuals who lose weight [7].

Sclerostin is a glycoprotein inhibitor of Wnt *ß*-catenin signaling pathway produced by the SOST gene and secreted mainly by osteocytes. Thus, it inhibits bone formation and may increase the risk of falls, fractures, osteopenia, and osteoporosis [8]. Sclerostin has also been detected in the vascular smooth muscle and is increased during the atherosclerosis process, limiting cardiovascular remodeling. This suggests a local upregulation of the action of sclerostin on arterial remodeling, which is of particular importance as the anti-sclerostin antibodies with prolonged pharmacokinetic release are being developed for the treatment of osteoporosis [9].

Recent studies have shown that resistance exercises increase muscle mass and can positively influence bone density. The mechanism to explain that is, however, still not completely understood. It's reported that sclerostin levels can be regulated by mechanical stimulation [7]. Therefore, the effect of resistance exercises on the inhibition of sclerostin to improve muscle mass and bone density has been studied. In this context, sclerostin has been suggested to be a good biomarker in detecting bone metabolism disorders [10, 11].

Serum sclerostin levels can be related to age, BMI, bone density, kidney function, and diabetes. The increase in life expectancy has also led to an increase in chronic diseases such as diabetes and CKD, as well as to the inherent complications of old age such as sarcopenia. It has, therefore, become important to study the relationship between biomarkers, like sclerostin, and high prevalence comorbidities, which have elevated morbidity and mortality and which lead to decreased life quality and increase the costs to health systems worldwide. The aim of this study is to determine the prevalence in patients undergoing hemodialysis of sarcopenia and its association with diabetes and with sclerostin levels.

## 2. Study Population and Methods

### 2.1. Patients

The study included 92 patients who were on hemodialysis at Agamenon Magalhães Hospital. All participants gave written informed consent. Individuals with decompensated hypo- and hyperthyroidism were excluded, as were those using systemic glucocorticoids and those with active neoplasia, with the exception of basal cell carcinoma.

The patients filled in a questionnaire and underwent a physical examination followed by an evaluation of muscle strength and physical performance.

### 2.2. Handgrip Strength and Physical Performance

Muscle strength was measured before sessions of hemodialysis using an e-clear manual digital dynamometer (model EH101), noting the average of three attempts made in the hand opposite to the arm with arteriovenous fistula taken at one-minute intervals. For patients using only venous catheters for hemodialysis, the test was carried out on both arms, the value taken into account being that of the dominant arm. Values of less than 30 kg were considered to be low muscle strength for men and values below 20 kg were considered to be low muscle strength for women. Physical performance was analyzed using a gait speed test of m/s along four meters. A result of less than 0.8 m/s was considered low for both men and women.

After hemodialysis session, the following anthropometric measures were recorded during the initial evaluation of the patient: weight; height; body mass index (BMI); abdominal circumference; hip circumference; arm and calf circumference.

Skeletal muscle mass index (SMI), muscle strength, and physical performance were evaluated in the diagnosis of sarcopenia. The diagnosis and classification of sarcopenia were carried and subdivided into pre-sarcopenia (low SMI), sarcopenia (low SMI plus low muscle strength or low physical performance), and severe sarcopenia (low SMI associated with low muscle strength and physical performance). SMI was calculated by dividing estimated skeletal muscle mass by squared height. Values of 10.75 kg/m^2^ or less in men and 6.75 kg/m^2^ or less in women were considered to be low [1].

### 2.3. Measurements

Body composition was performed using multifrequency electric bioimpedance (Biodynamics 310, UK) immediately after hemodialysis, that is, with a weight considered to be “dry”.

Fat-free mass index was calculated by dividing fat-free body mass by squared height.

Blood samples for serum sclerostin measurements were collected from 83 individuals immediately before hemodialysis, utilizing the patients' venous access used in dialysis. These samples were analyzed using the sandwich quantitative method ELISA (of the Biomedica Co., Vienna, Austria). The coefficients of variation intra- and interassay were 5% and 3%, respectively. Values above 31 pmol/L were considered to be elevated. Samples were also taken on this occasion to measure total cholesterol, HDL, LDL, triglycerides, predialysis glucose, calcium, phosphorus, PTH, and 25OHD.

The study was designed in accordance with the Declaration of Helsinki and was approved by the Ethics in Research Committee of the Agamenon Magalhães Hospital (CAAE 52567615.6.0000.5197).

### 2.4. Statistical Analysis

Data collected from each individual was entered into a data input mask created through use of Epilnfo version 7 software and after any necessary corrections was exported to the Statistical Package for the Social Sciences, version 13.0, so that statistical analysis concerning the objectives of this study could be carried out.

To begin with, and in order to characterize the population researched, a descriptive analysis of data was carried out which obtained absolute frequencies (*N*) and relative frequencies (%) with respect to the variables of qualitative/categorical interest and, in the case of quantitative/numerical variables, they were presented in the form of mean ± standard deviation.

For a univariate analysis, with the objective of identifying differences between the groups being compared according to the variables of the study, the Chi-squared test was used (or the Fisher test when necessary) for qualitative variables, and Student's *t-*test was used to compare groups considered in relation to quantitative variables. With the objective of verifying the association of sclerostin with continuous variables of the study, the Pearson coefficient of correlation with a respective *p* value was used.

Variables which present some association with the outcome in the univariate analysis (value of *p* < 0.20) were selected to form the initial model of multivariate analysis in which logistic models were adjusted. Subsequently, the selection for a final model using *backward* model was carried out. The variables which present some association (value of *p* < 0.05) with the variables sarcopenia, sclerostin, and diabetes remained in the final model. Besides this, the *odds ratio* (OR), which represents the odds that an outcome will occur, was used as a risk measure and obtained together with its respective confidence intervals (95% CI).

## 3. Results

Mean age was 63.3 ± 13.6 years (63% men, 41% with diabetes). The most prevalent comorbidity was arterial systemic hypertension (88%) followed by diabetes (44.6%) (Table 1). Of the 41 individuals suffering from mellitus diabetes, two were diagnosed with type 1 diabetes and 39 with type 2 diabetes.

A low muscle mass index was identified in 65.2% of the individuals studied (76.7% were male and 36.7% diabetics), of which 10.9% were at the pre-sarcopenia stage, 23.9% had sarcopenia, and 30.4% had severe sarcopenia.

The walking speed test showed significant differences between the sexes, with 51% of women presenting low stress levels (*p*=0.002). The muscle strength test, however, showed no significant statistical difference between the sexes (*p*=0.251).

The average level of serum sclerostin among those studied was 86.9 ± 39 pmol/L, showing higher levels for men than for women (94.6 ± 41.7 vs. 73.8 ± 30.3; *p*=0.017).

The differences between subjects with and without diabetes were as follows: pre-sarcopenia (4.9% vs. 15.7%); sarcopenia (17.1% vs. 29.4%); and severe sarcopenia (31.7% vs. 29) (*p*=0.080). An impaired walking speed was identified in 61% of the diabetics and 51% of those patients without diabetes. Reduced muscle strength was observed in 80.5% of diabetics and 64.7% of those without diabetes (Table 2).

Serum sclerostin levels presented a negative association with muscle mass index, while a positive association was observed between the presence of diabetes and sclerostin levels. Serum sclerostin levels were significantly higher in individuals with low muscle mass index (94.9 ± 40.7; *p* < 0.001) and in those individuals with diabetes (97.2 ± 46.6; *p* < 0.003) (Table 3). In the univariate analysis, high levels of sclerostin were also significant when diabetics with low lean body mass were analyzed (114.5 ± 51.7; *p*=0.015).

Fat-free mass index was positively associated with muscle strength in men (*p*=0.036). However, age was negatively associated with this index.

## 4. Discussion

This study determined the prevalence of sarcopenia in patients undergoing hemodialysis and evaluated the association in these patients of diabetes and concentrations of serum sclerostin. The evaluation of muscle strength, physical performance, and muscle mass measurement showed that 31.7% and 29.4% of patients, with and without diabetes, respectively, had sarcopenia. Kim et al. [12] evaluated 95 patients undergoing hemodialysis and observed that they had a mean age of 63.9 ± 10.0, 57.2% were male, and 67% had diabetes. A study carried out to estimate the prevalence in conservative treatment of sarcopenia in patients with chronic kidney disease and its association with mortality reported that 53% patients were over 60 years of age, 62% were male, and 49% had diabetes [13].

In this sample of individuals with a mean age of 56.8 ± 15.6 years, our study identified low muscle mass index and sarcopenia in 65.2% and 23.9% of patients, respectively, with a greater prevalence in men than in women (32.8% vs. 8.8%), and identified diagnosis of diabetes in 17.1% of the individuals. Pereira et al. [14] in their study using bioimpedance and dynamometer to diagnose sarcopenia found in a group whose average age was 60.3 ± 11.9, and of whom 82.4% were men, a prevalence of sarcopenia in 5.9% of patients and diabetes in 35.3% of patients. However, the differences found in the prevalence of sarcopenia are probably related to the fact that our study evaluated individuals with chronic kidney disease already undergoing hemodialysis and to the fact that we used for sarcopenia diagnostic criteria not only the association of low muscle mass index with reduced muscle strength, but also the association of low muscle mass with a reduced walking speed. A Korean study carried out on 810 individuals found a prevalence of sarcopenia of 15.7% of them by use of total body densitometry and showed that diabetics have three times more chance of having sarcopenia as compared to those who do not have diabetes, something which reinforces data that diabetes predisposes sarcopenia [15].

Corroborating with our findings, a study carried out in three hemodialysis centers in Korea following EWGSOP criteria, using bioimpedance and dynamometer, found a prevalence of 9.5% and 33.7% of pre-sarcopenia and sarcopenia, respectively [16]. A Chinese study showed different results for pre-sarcopenia (14.4%) and sarcopenia (14.8%). Although this study also used bioimpedance and dynometer to diagnose sarcopenia, different parameters were used, based on criteria defined by the Asian Working Group for Sarcopenia (AWGS) [15].

This study showed that impaired walking speed and muscle strength were more prevalent in the group of diabetics than in the group of non-diabetics (61% vs. 51% and 80.5% vs. 64%, respectively), but without significant difference in the univariate analysis. In the multivariate analysis carried out on the general public, we observed that women have 4.3 times more chance of presenting a low walking speed than men (IC 95% 1.663–11.066; *p* < 0.003). Wang et al. [17] in their study failed to identify any significant difference in muscle strength between men and women in comparing diabetics with the control group. In the walking speed test, however, they noted a significant difference between men (1.08 ± 0.22 vs. 1.23 ± 0.18, *p* < 0.001) and women (1.07 ± 0.26 vs. 1.26 ± 0.16, *p* < 0.001). Similar data was found in a study within a control group comparing physical function of women without chronic kidney disease who had mellitus diabetes type 2 (12.4 ± 5.9 kg vs. 13.4 ± 5.3 kg; *p*=0.086) [13].

Our study detected an average of 86.9 ± 39.0 pmol/L in levels of serum sclerostin in the group studied, being higher for men than for women (94.6 ± 41.7 vs. 73.8 ± 30.3). After adjustments for potential confounders, serum sclerostin levels present a negative association with muscle mass index (*p* < 0.001) and a positive association with diabetes (*p*=0.003). The average of serum sclerostin levels was significantly higher in the group of diabetics (97.2 ± 46.6 vs. 79.7 ± 31.2; *p* 0.044), mainly among men (109.5 ± 50.9 vs. 77.4 ± 31.2; *p* < 0.044). A study of diabetics undergoing hemodialysis presents high levels of sclerostin compared to those without diabetes [18]. Regarding patients with diabetes not on dialysis, other studies have shown increased sclerostin in comparison to the non-diabetic population [19–21], and this may explain, at least in part, the mechanisms underlying bone fragility in diabetics.

Kim et al. [12] in their study evaluated a group of 302 diabetics treated in clinics of endocrinology and nephrology in two Korean hospitals and distributed their sample in groups defined by levels of glomerular filtration. They found elevated levels of serum sclerostin in diabetics, with men having a significantly higher level than women (121.4 ± 6.8 vs. 76.1 ± 6.5 pmol/L, *p* < 0.001). Sclerostin levels were higher for those patients with the worst glomerular filtration rates, emphasizing the relationship between sclerostin and kidney function. This data is in agreement with the findings of Pelletier et al. [18], that sclerostin levels were higher for individuals with chronic kidney disease than for the individuals of the control group.

Serum sclerostin levels are negatively and significantly consistent with low muscle mass (94.9 ± 40.7; *p* < 0.001). This can be justified by studies which show that sclerostin is regulated by mechanical stimulation. Robling et al. [22] in their study investigated sclerostin regulation promotion of a mechanically stimulated and disused environment for mice. The conclusion of the study was that sclerostin levels were reduced by mechanical stimulation and were intimately related to the distribution of tissue tension, which consequently improved bone formation.

Fat-free mass index is positively associated with muscle force in men, while age is negatively associated with this index.

Regarding sclerostin and serum PTH concentrations, we did not find significant correlations between them and this is in agreement with a previous study carried out by [15, 18] in which no significant difference of PTH in individuals with kidney dysfunction not undergoing hemodialysis was found. In contrast, Cekja et al. [23], in their study, found that sclerostin was negatively correlated with PTH (*ρ*=−0.34, *p*=0.01). Our patients with diabetes showed higher sclerostin concentrations than non-diabetic patients and significantly higher values for adiposity measures such as BMI, WC, and HC. For another area of interest, a study in a health population demonstrated a positive association between BMI with android and gynoid fat and sclerostin [24].

By contrast, the ARCH study, which evaluates the comparison between Romosozumab (a monoclonal antibody which inhibits sclerostin) and Alendronate in the prevention of bone fractures in women with osteoporosis, although showing a higher and faster gain in bone mass and a reduction in hip fractures, observed a higher prevalence of cardiovascular events (2.5% vs. 1.9%). In a double blind study, cardiovascular events were more common in the group taking Romosozumab than in the group taking Alendronate, with ischemic cardiac and cerebral events contributing to this imbalance [25].

There were more serious adverse cardiovascular events in this study than in the FRAME study (which evaluates the existence of vertebral fractures in women with osteoporosis who took only Romosozumab for one year), [26] the incidence of such events being balanced in the group taking Romosozumab and that taking a placebo. A possible underlying mechanism for these events could be the role of sclerostin in vascular smooth muscle, as studies have shown that SOST is present in other tissues, including the aorta vascular smooth muscle. The inhibition of sclerostin by taking Romosozumab could thus alter vascular remodeling, which is normally induced through the Wnt pathway. Besides this, sclerostin is positively regulated in areas of vascular calcification. Another possibility, although remote, is that the compared drug Alendronate is cardioprotective, and therefore the number of cardiovascular events in the Romosozumab group seems to be relatively higher than expected [27].

## 5. Conclusion

We found higher serum sclerostin concentrations in hemodialysis patients with diabetes which were inversely related to muscle mass.

## Figures and Tables

**Table 1:** Clinical characteristics of the study patients.

Variable	Patients		*p* value
	With diabetes	Without diabetes	
Gender			
Male	26 (63.4%)	32 (62.7%)	
Female	15 (36.6%)	19 (37.3%)	0.947
Age (yr)	64.3 ± 10.3	62.5 ± 15.8	0.522
Diabetes duration (yr)			
3–5 years	1 (2.4%)	0 (0.0%)	
>5 years	40 (97.6%)	0 (0.0%)	—
Time since diagnosis of CKD			
<3 years	17 (41.5%)	11 (21.6%)	
3–5 years	6 (14.6%)	9 (17.6%)	
>5 years	18 (43.9%)	31 (60.8%)	0.117
Time in hemodialysis			
<3 years	31 (75.6%)	30 (58.8%)	
3–5 years	6 (14.6%)	9 (17.6%)	
>5 years	4 (9.8%)	12 (23.5%)	0.168
Systolic pressure (mmHg)	146.3 ± 21.3	136.3 ± 21.5	**0.028**
Diastolic pressure (mmHg)	70.2 ± 10.8	69.4 ± 14.8	0.756
Weight (kg)	74.3 ± 14.8	68.0 ± 11.7	**0.024**
Height (cm)	167.1 ± 7.2	168.0 ± 9.7	0.598
BMI (kg/m^2^)	26.6 ± 5.2	24.1 ± 3.7	**0.010**
WC (cm)	103.1 ± 12.2	94.9 ± 10.7	**0.001**
HC (cm)	105.0 ± 10.3	100.2 ± 7.1	**0.012**
WHR	1.0 ± 0.1	0.9 ± 0.1	**0.021**
Serum sclerostin (pmol/l)	97.2 ± 46.6	79.7 ± 31.2	**0.044**

CKD, chronic kidney disease; BMI, body mass index; WC, circumference waist; HC, hip circumference; WHR, waist hip ratio.

**Table 2:** Body composition and laboratory data.

Variable	With diabetes	Without diabetes	*p* value
Fat mass (%)	29.0 ± 8.5	27.9 ± 7.2	0.507
Fat-free mass (kg)	52.2 ± 9.7	48.9 ± 9.6	0.103
Muscle mass (kg)	25.3 ± 6.1	23.9 ± 6.2	0.270
SMI (kg/m^2^)	9.2 ± 2.5	8.4 ± 1.8	0.074
Gait speed (m/s)	0.8 ± 0.3	0.8 ± 0.3	0.282
Handgrip strength (kg)	20.4 ± 7.1	22.5 ± 8.3	0.202
Predialysis FPG (mg/dL)	182.2 ± 102.5	104.3 ± 28.1	**<0.001**
Total cholesterol (mg/dL)	153.4 ± 45.3	169.3 ± 39.8	0.077
HDL-C (mg/dL)	42.2 ± 10.5	48.8 ± 18.8	**0.035**
LDL-C (mg/dL)	75.2 ± 30.6	91.1 ± 37.4	**0.035**
Triglycerides (mg/dL)	158.2 ± 90.6	152.6 ± 87.9	0.764
PTH (pg/mL)	293.2 ± 255.5	387.1 ± 299.2	0.114
Calcium (mg/dL)	9.1 ± 0.6	9.3 ± 0.8	0.164
Phosphorus (mg/dL)	5.5 ± 1.8	4.9 ± 1.6	0.091
25OHD (ng/mL)	27.8 ± 4.4	29.2 ± 5.8	0.473

Data is expressed as mean ± SD. DM, diabetes mellitus; SMI, skeletal muscle mass index; FPG, fasting plasma glucose; HDL-C, high-density lipoprotein cholesterol; LDL-C, low-density lipoprotein cholesterol; PTH, parathyroid hormone; 25OHD, 25-hydroxyvitamin D; SD, standard deviation.

**Table 3:** Multivariate analysis showing the independent association of sclerostin with low skeletal muscle index and the presence of diabetes.

Variables	*N*	Serum sclerostin (pmol/L)	OR^*∗*^	95% CI	*p* value
SMI					
Normal	29	71.9 ± 31.0	1.000	—	
Low	54	94.9 ± 40.7	1.427	1.186–1.717	**<0.001**
Diabetes					
No	49	79.7 ± 31.2	1.000	—	
Yes	34	97.2 ± 46.6	1.328	1.110–1.589	**0.003**

Data are expressed as mean ± SD. OR, odds ratio; CI, confidence interval; SMI, skeletal muscle mass index. ^*∗*^OR adjusted by the effect of other confounders.

## Data Availability

The data used to support the findings of this study are available from the corresponding author upon request.
